# A Multi-Electrode Pixel Structure for Quick-Response Electrowetting Displays

**DOI:** 10.3390/mi13071103

**Published:** 2022-07-14

**Authors:** Lixia Tian, Shufa Lai, Taiyuan Zhang, Wei Li, Biao Tang, Guofu Zhou

**Affiliations:** Guangdong Provincial Key Laboratory of Optical Information Materials and Technology & Institute of Electronic Paper Displays, South China Academy of Advanced Optoelectronics, South China Normal University, Guangzhou 510006, China; 2019010238@m.scnu.edu.cn (L.T.); taiyuan.zhang@guohua-oet.com (T.Z.); wei.li@guohua-oet.com (W.L.); b.tang@m.scnu.edu.cn (B.T.); guofu.zhou@m.scnu.edu.cn (G.Z.)

**Keywords:** electrowetting display, multi-electrode, pixel structure, sub-electrode, response time, aperture ratio, simulation

## Abstract

A new reflective display technology, the electrowetting display (EWD), has the advantages of high contrast ratio, high reflectivity, and ultralow power consumption. The response speed of EWDs has an important influence on optical performance, and pixel structure is one of the key factors affecting the response speed of EWDs. In order to improve the response speed, a new multi-electrode pixel structure is proposed in this paper. This structure was realized by dividing the pixel into four square-shaped sub-electrodes, and a three-dimensional EWD simulation model was established. In this model, a driving voltage was first applied to one of these sub-electrodes, and the oil was ruptured. Then, its two adjacent sub-electrodes were also supplied with driving voltages, so as to spur the oil to move to a pixel corner quickly. Simulation results showed that the response speed of EWDs can be effectively improved by using the proposed multi-electrode pixel structure. Compared with a single-electrode pixel structure, the oil rupture response time of the multi-electrode pixel structure was advanced by 0.6 ms. The pixel achieved a 2.7 ms faster response time than the single-electrode pixel for reaching a 50% aperture ratio in an opening process, and the maximum aperture ratio was increased by 6.2%.

## 1. Introduction

A new reflective paper-like display technology, the electrowetting display (EWD), was first proposed by Robert Hayes in 2003 [1]. By applying an external electric field on oil in pixels, EWDs can realize an optical switch and have the ability of video-speed display applications [2,3]. However, due to the limitation of the principle and structure of EWDs, there are still many technical defects, such as oil backflow, oil dispersion, charge trapping, the hysteresis effect, and other problems, which need to be optimized urgently [4]. Scholars have carried out relevant research on improving response speed of EWDs.

In a driving process, driving waveforms directly affect the display effect of EWD pixels [5]. Yung [6] designed an asymmetrical alternating polarity driving scheme, which reduced the response time of EWDs. The asymmetry in driving energy was the difference between an alternating polarity signal with symmetrical energy and a common signal with variable direct current (DC) voltage. The objective of the asymmetrical driving energy was used to provide a consistent electric field on EWDs with the alternating polarity method, so as to improve the charge-trapping phenomenon and save response time. Oil motion control is a key to affect the optical performance of EWDs. Yi [7] used an amplitude-frequency mixed-modulation method to drive EWDs, so that oil quickly reached a target reflectivity. The response time was reduced by 70% compared with that of a conventional pulse width modulation (PWM) scheme. Further, Hsieh [8] established a three-dimensional model to study hydrodynamic behavior and to explore the influence of materials and interfaces on EWD performance. When the wetting contact angle was set to 90° and applied with a voltage of 40 V, the response time was effectively improved in both the opening stage and the closing stage. Moreover, a simple electrowetting dynamic model was proposed by Zhou [9] to study the dynamic response of oil by describing “on” and “off” in pixels. It pointed out that there was an optical response delay during an on-switching process and an optical response asymmetry between “on and off” switching and that oil rearrangement time in the on-switching stage was relatively long. Usually, a rearrangement stage was the most time-consuming and it dominated the pixel opening time. In addition, the pixel structure was also an important factor that could affect the response speed. Andrea Giraldo [10] proposed a pixel structure with a hydrophilic patch or a staircase. By changing the architecture of pixels, hysteresis-free pixel switching and high control of fluid movement were achieved. Pixels with hydrophilic patches showed quick responses in both the “on and off” stages. The electric field strength was the highest in the vicinity of the hydrophilic patch when the pixel was opened. This method eliminated the hysteresis effect from manufacturing process, but it affected the pixel aperture ratio. Dou [11,12] introduced an extra pinning structure (EPS) into EWD pixel structure to achieve precise control of oil rupture position and movement direction. The localized pinning and capillary effects effectively guided the oil contraction direction and significantly accelerated the pixel opening process. Therefore, improvement of pixel structure is helpful to increase the response speed of EWDs.

In order to improve the response speed, a multi-electrode pixel structure with four sub-electrodes is proposed in this paper, and a three-dimensional dynamic simulation model was established. Furthermore, a sequence of driving voltage is applied to evaluate the performance of the proposed pixel structure model.

## 2. Principles of EWDs

**Figure 1:**
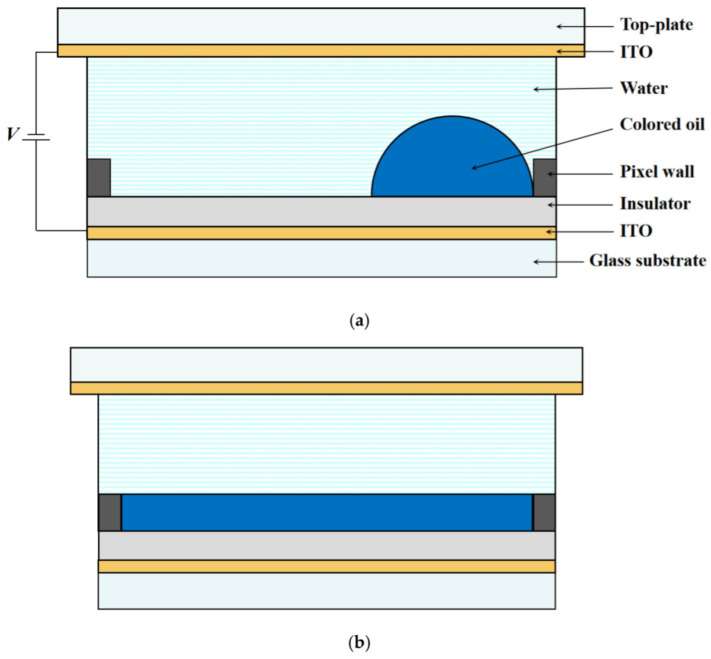
Pixel structure and operating principles of EWDs. The optical stack consists of a top plate, a glass substrate, indium tin oxide (ITO) electrodes, a hydrophobic insulator layer, pixel wall, colored oil, and water. (**a**) With an applied voltage, oil is shrunk to a corner and the pixel is in a white on-state. (**b**) With no voltage applied, oil spreads uniformly and the pixel is in a colored off-state.

Electrowetting display technology refers to a shrinkage and spreading process of colored oil inside a pixel by applying a voltage between a top and bottom substrate to change the wettability of the conductive liquid on a hydrophobic insulator layer [13,14]. A pixel structure of EWDs mainly consists of a top plate, a glass substrate, two indium tin oxide (ITO) electrodes, a hydrophobic insulator layer, pixel wall, colored oil, and water [15]. The pixel structure and operating principles of EWDs are shown in Figure 1. For a pixel, a complete switching response consists of “on and off” stages. When a voltage is applied, the volume force perpendicular to the oil lowers the oil and makes the water contact the hydrophobic insulator layer. After that, the electric field on the three-phase contact line is distorted, and oil moves toward a pixel corner under the combined action of electric capillary and volume force. In this stage, the aperture ratio of the pixel increases, and the contact angle between the conductive liquid and the hydrophobic insulator layer decreases. Finally, the electric field energy of the electrowetting system is balanced with the surface potential energy of oil. When the voltage is withdrawn, the electric field energy disappears. Then, the potential energy and surface energy gathered by oil spread freely. The hydrophobic insulator layer is covered by oil again, that is, the pixel is turned off. In this way, an optical switch is formed to realize a monochromatic display of the pixel [16].

**Figure 2:**
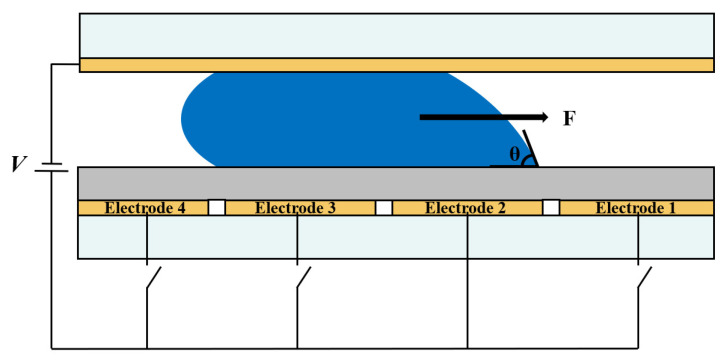
Schematic diagram of force on oil movement driven by electrowetting. By activating electrode 2, the contact angle θ is reduced and the droplet is deformed accordingly. The electric field force drives the whole droplet toward the right.

Electrostatic field force is a key factor affecting oil movement of EWDs [17,18]. The motion of oil is determined by electric field intensity. According to the principle of electrowetting, for an oil droplet, applying an electric field to only one side of the oil causes an imbalanced force on both sides of the oil, which drives the whole oil flow to one side [19,20,21]. Figure 2 shows a schematic diagram of oil movement by electrowetting. The bottom substrate contains a group of addressable electrodes. The contact angle is varied by the applied voltage, and the oil is deformed and shifted by electric field forces. The diagram shows that the contact angle θ decreases by activating electrode 2. Then, the oil deforms accordingly and moves to the right [22].

## 3. Numeric Methodology and Modeling

A dynamic model of the three-dimensional EWD was realized by numeric methodology. By simulating the transient motion of oil, including oil rupture, wetting, and recovery, hydrodynamic behavior of the pixel in “on and off” states could be accurately predicted [23]. COMSOL Multiphysics software was used to simulate the laminar two-phase flow under the action of electric field, and the laminar physical field was coupled with the phase field and the electrostatic field [24,25]. The finite element method (FEM) was used to solve the relevant governing equations to track topological changes in the oil–water interface in the model [8].

### 3.1. Numeric Methodology

The phase field is used to describe the dynamic change process of the two-phase flow interface, and the motion of the interface can be traced indirectly by solving the phase-field equation [26,27,28]. The phase-field equation is defined as Equations (1) and (2):

(1) $$\frac{\partial \varphi }{\partial t}+u\cdot \nabla \varphi =\nabla \cdot \frac{\gamma \lambda }{\varepsilon^{2}}\nabla \psi \;$$

(2) $$\gamma =\chi \varepsilon^{2}\;$$

where, φ is defined as a continuous phase-field variable, u represents the fluid velocity, and γ, λ and ψ denote the mobility, mixed energy density, and phase-field help variable, respectively. The mobility γ is a scalar, χ is the mobility tuning parameter, and ε  is a parameter that controls the interface thickness.

The mixed energy density λ helps to adjust the interface thickness according to the surface tension of the modeled interface. The relationship between the mixed energy density and the surface tension coefficient σ is described in Equation (3).

(3) $$\sigma =\frac{2\surd 2}{3}\frac{\lambda }{\varepsilon }$$

In the phase-field method, the phi derivative of external free energy $\frac{\partial f_{ext}}{\partial \varphi }$ is set to zero based on the principle of system energy minimization. Therefore, the phase-field help variable is finally defined as Equation (4).

(4) $$\psi =-\nabla \cdot \varepsilon^{2}\nabla \varphi +\left(\varphi^{2}-1\right)\varphi +\left(\frac{\varepsilon^{2}}{\lambda }\right)\frac{\partial f_{ext}}{\partial \varphi }$$

The confined EWD model has two fluids, water and oil, which are assumed to be incompressible and mutually immiscible Newtonian fluids [29]. In order to describe the dynamic change process of the immiscible two-phase flow, the transmission of mass and momentum is controlled by the incompressible Navier–Stokes equation, as shown in Equation (5):

(5) $$\rho \left(\frac{\partial u}{\partial t}+u\cdot \nabla u\right)=-\nabla p+\nabla \cdot \left(\mu \left(\nabla u+{\left(\nabla u\right)}^{T}\right)-\frac{2}{3}u\left(\nabla \cdot u\right)I\right)+F$$

(6) $$F=F_{st}+\rho g+F_{vf}$$

where, u is the flow velocity, and p, ρ, and μ represent the flow pressure, density, and hydrodynamic viscosity, respectively. Each item in Equation (5) corresponds to inertia force, pressure, viscous force, and external force acting on the flow. The external force is mainly composed of surface tension $F_{st}$, gravity ρg, and volume force $F_{vf}$. The Navier–Stokes equation for momentum conservation and the continuity equation for mass conservation need to be calculated simultaneously for the laminar flow field, and the continuity equation is defined as Equation (7).

(7) $$\frac{\partial u}{\partial t}+\nabla \cdot \left(\rho u\right)=0$$

Electrostatic field force is a main factor in flowing change of fluid, and is caused by the electric field gradient formed by a nonuniform electric field. For incompressible fluids, the electrostrictive density can be ignored and the electrostatic volume force $F_{evf}$ can be calculated by the divergence of the Maxwell stress tensor (MST) [8], which is described by Equation (8):

(8) $$F_{evf}=\nabla T_{ij}$$

where, $T_{ij}$ is the tensor. The MST equation is shown in Equation (9):

(9) $$T_{ij}=ED^{T}-\frac{1}{2}\left(D\cdot EI\right)$$

where I is an identity matrix, E is an electric field strength, and D is an electric flux density. The electric flux density can be defined as Equation (10), where $\varepsilon_{0}$ and $\varepsilon_{r}$ represent the permittivity of free space and relative permittivity, respectively.

(10) $$D=\varepsilon_{0}\varepsilon_{r}E$$

In the three-dimensional simulation model, the MST obtains a matrix form, as shown in Equation (11).

(11) $$T=\left[\begin{array}{ccc} T_{xx} & T_{xy} & T_{xz}\\ T_{yx} & T_{yy} & T_{yz}\\ T_{zx} & T_{zy} & T_{zz} \end{array}\right]$$

By substituting parameters, the matrix form can be expressed in Equation (12).

(12) $$T=\left[\begin{array}{ccc} \varepsilon_{0}\varepsilon_{r}E_{x}^{2}-\frac{1}{2}\varepsilon_{0}\varepsilon_{r}\left(E_{x}^{2}+E_{y}^{2}+E_{z}^{2}\right) & \varepsilon_{0}\varepsilon_{r}E_{x}E_{y} & \varepsilon_{0}\varepsilon_{r}E_{x}E_{z}\\ \varepsilon_{0}\varepsilon_{r}E_{x}E_{y} & \varepsilon_{0}\varepsilon_{r}E_{y}^{2}-\frac{1}{2}\varepsilon_{0}\varepsilon_{r}\left(E_{x}^{2}+E_{y}^{2}+E_{z}^{2}\right) & \varepsilon_{0}\varepsilon_{r}E_{y}E_{z}\\ \varepsilon_{0}\varepsilon_{r}E_{x}E_{z} & \varepsilon_{0}\varepsilon_{r}E_{y}E_{z} & \varepsilon_{0}\varepsilon_{r}E_{z}^{2}-\frac{1}{2}\varepsilon_{0}\varepsilon_{r}\left(E_{x}^{2}+E_{y}^{2}+E_{z}^{2}\right) \end{array}\right]$$

Performing the Jacobian on the MST, the volume force is obtained, as shown in Equation (13).

(13) $$F=\left[\begin{array}{ccc} \frac{\partial \left(T_{xx}\right)}{\partial x} & \frac{\partial \left(T_{xy}\right)}{\partial y} & \frac{\partial \left(T_{xz}\right)}{\partial z}\\ \frac{\partial \left(T_{yx}\right)}{\partial x} & \frac{\partial \left(T_{yy}\right)}{\partial y} & \frac{\partial \left(T_{yz}\right)}{\partial z}\\ \frac{\partial \left(T_{xz}\right)}{\partial x} & \frac{\partial \left(T_{yz}\right)}{\partial y} & \frac{\partial \left(T_{zz}\right)}{\partial z} \end{array}\right]$$

### 3.2. Boundary Conditions

Boundary conditions are a prerequisite for the governing equation to obtain a definite solution on the region boundary. When the Laplace equation is used to solve the electrostatic field, the boundary condition around the model is set to zero charge, except for the electrode region. For the electrode area, the top substrate is grounded and the voltage at the bottom of the hydrophobic insulator layer is specified. Boundary conditions of solid surfaces were set to wetted walls, including the surface of the hydrophobic insulator layer, the pixel wall, the hydrophilic grid, and the top substrate. The contact angle of the solid surface is defined as the contact angle with water $\theta_{w}$. The wetted wall boundary condition is determined by Equations (14) and (15) [30]:

(14) $$n\cdot \varepsilon^{2}\nabla \varphi =\varepsilon^{2}\cos\left(\theta_{w}\right)\left|\nabla \varphi \right|$$

(15) $$n\cdot \frac{\gamma \lambda }{\varepsilon^{2}}\nabla \psi =0$$

where n is an unit vector perpendicular to the wall, and the two-phase flow contact interface is chosen as the initial interface location, i.e., φ=0. These four sides of the pixel in the model are chosen as the inlet and outlet boundary conditions, which are set as the outlet end, and the pressure constraint term p=0 is set at the end point. In addition, wall condition is set to no sliding, bottom contact surface is set as Navier sliding, and initial value of fluid velocity is set to 0 m/s.

Parameters used in the simulation model are listed in Table 1. The fluid in the model was set as an incompressible flow. For the current structure, the effect of pressure on the dynamic viscosity was neglected. During the movement of the fluid, it was assumed that the temperature (25 °C) remained constant, then the thermal expansion of the fluid was neglected.

## 4. Results and Discussion

### 4.1. Multi Electrode Pixel Structure Model Design

**Figure 3:**
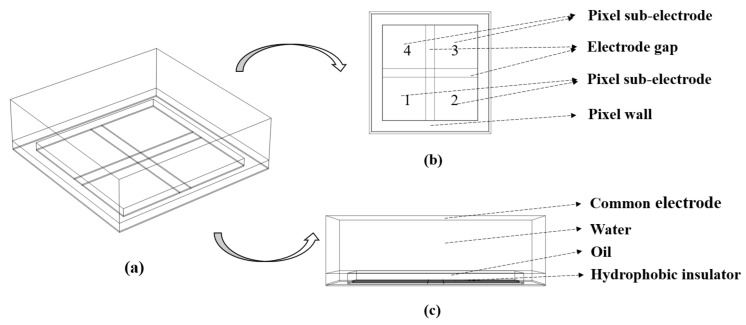
Multi-electrode pixel structure model diagram of an EWD pixel. (**a**) Three-dimensional simulation model of a multi-electrode structure in a pixel. The pixel was divided into a field shape with four sub-electrodes. (**b**) Top view of the multi-electrode pixel structure. (**c**) Side view of the multi-electrode pixel structure.

In this paper, COMSOL Multiphysics was used to build a three-dimensional EWD simulation model, and a pixel electrode structure with four sub-electrodes was designed, as shown in Figure 3. This structure was realized by dividing the pixel into four square-shaped sub-electrodes. The top substrate contained a continuous common electrode, while the bottom substrate contained a set of addressable electrodes.

**Figure 4:**
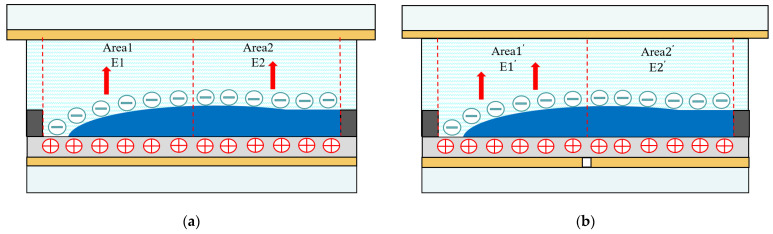

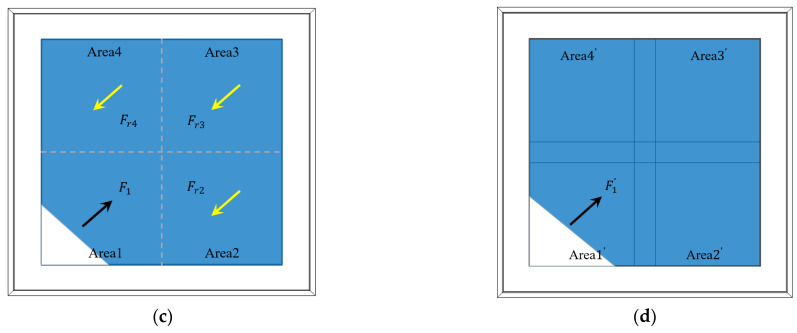
Comparison between the internal electric field and electric field force of pixels. (**a**) Cross-sectional view of the internal electric field distribution when voltage was applied to a single-electrode pixel. (**b**) Cross-sectional view of the internal electric field distribution when voltage was applied to a multi-electrode pixel in time sequence. (**c**) The direction of electric field force when oil in Area 1 of the single-electrode pixel was deformed by the electric field force. (**d**) The direction of electric field force when oil in $\mathrm{Area}\;1^{’}$ of the multi-electrode pixel was deformed by the electric field force.

As shown in Figure 4, it was assumed that the internal space of a single-electrode pixel was divided into four regions, namely, Area 1, Area 2, Area 3, and Area 4. E1 and E2 respectively represented the internal electric fields formed by Area 1 and Area 2. Similarly, the internal space of the multi-electrode pixel was divided into $\mathrm{Area}1^{’}$, $\mathrm{Area}2^{’}$, and $\mathrm{Area}3^{’}$, and $\mathrm{Area}4^{’}$. $E1^{’}$, and $E2^{’}$ represented the internal electric field formed in $\mathrm{Area}\;1^{’}$, and $\mathrm{Area}\;2^{’}$. Red arrows indicate the direction of the internal electric field, black arrows indicate the force direction in each area, and yellow arrows indicate the resistance direction under the electric field force in other areas.

For a single-electrode pixel structure, the oil began to move when voltage V was applied. The change in the internal electric field is shown in Figure 4a. $F_{1}$ represents the electric field force on Area 1. For Area 1, where the oil initial rupture was located, the oil in Area 1 was also subjected to static resistance caused by the electric field force in Area 2, Area 3, and Area 4, which were respectively recorded as $\;F_{r2}$, $F_{r3}$, and $F_{r4}$. Therefore, within a limited time, the combined electric field force on oil in Area 1 was defined as $F=F_{1}-F_{r2}-F_{r3}-F_{r4}$.

In a multi-electrode pixel structure, when the electrode in $\mathrm{Area}1^{’}$ was first applied with voltage, the change in the internal electric field was shown in Figure 4b. $F_{1}^{’}$ was the electric field force in $\mathrm{Area}1^{’}$, and the electric field force was concentrated on this sub-electrode, which would not be subject to the resistance caused by the application of electric fields from other areas. Therefore, the combined electric field force on oil in $\mathrm{Area}1^{’}$ could be defined as $F^{’}$=$F_{1}^{’}$ in the restricted time. In this case, the electrostatic field force was able to further squeeze and push oil. This was verified by numeric simulation.

**Figure 5:**
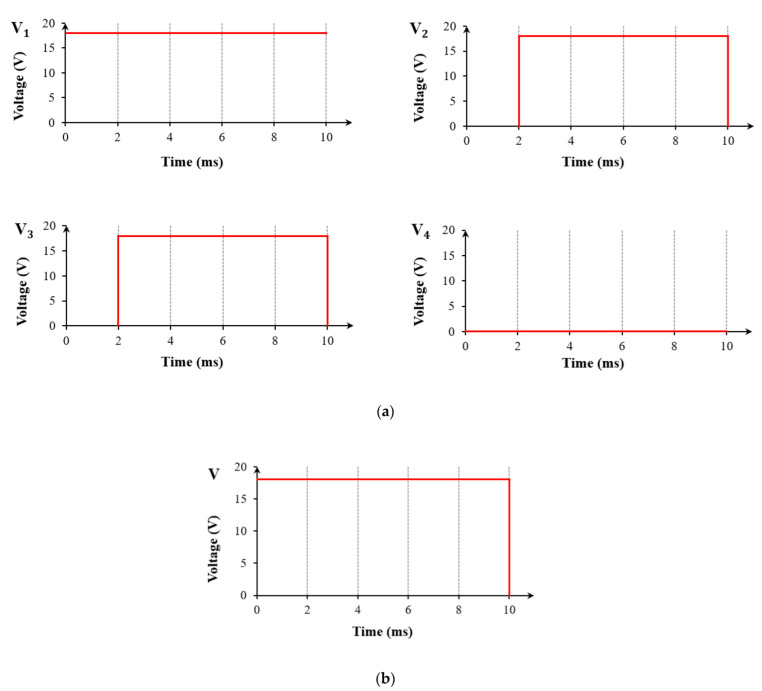
Sequence diagram of applying voltage to a multi-electrode pixel and a single-electrode pixel. (**a**) Sequence diagram of applying voltage to electrodes of the multi-electrode pixel. V1, V2, V3, and V4 represent the voltage applied to sub-electrodes 1, 2, 3, and 4, respectively. The voltage was first applied to sub-electrode 1, then simultaneously applied to its two adjacent sub electrodes 2 and 4, the finally removed. (**b**) Sequence diagram of applying voltage to electrode of the single-electrode pixel. The voltage was applied to the whole pixel and then removed.

In order to induce oil to rupture and shrink rapidly to a specified corner, it was necessary to apply driving voltages to sub-electrodes of the multi-electrode pixel structure sequentially. Figure 5a shows the sequence diagram of applying voltage to sub-electrodes. First, a driving voltage was applied to one sub-electrode to make oil rupture and move quickly. Then, voltages were applied to the two adjacent sub-electrodes simultaneously, which made the oil advance quickly to the designated corner. The purpose of continually applying voltage to the first sub-electrode was to prevent oil backflow. Finally, voltages were removed, and oil reverted to the state before driving. Figure 5b shows the sequence of applying voltage to the electrode of a single-electrode pixel structure. A voltage was applied to the whole pixel and then withdrawn.

### 4.2. Effects of Pixel Electrode Structure on Response Speed and Aperture Ratio

**Figure 6:**
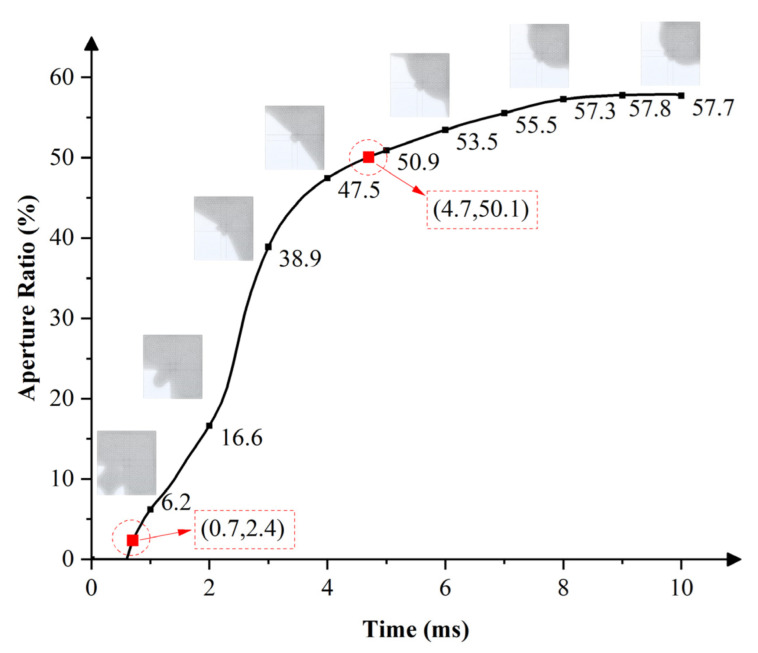
The switch-on response curve of EWDs with a multi-electrode pixel structure. Oil ruptured in the area of sub-electrode 1 and moved rapidly toward the pixel corner. With the voltage applied to two adjacent sub electrodes, oil was further accelerated to move to the specified pixel corner. The oil ruptured at 0.7 ms and the pixel reached a maximum aperture ratio of 57.9% at 9.5 ms. It took only 4.7 ms to reach an aperture ratio of 50%.

An 18 V current was applied to the multi-electrode pixel according to the voltage sequence diagram in Figure 5a, oil shrunk under the action of electrostatic field force, and the response curve of the pixel opening process is shown in Figure 6. The oil began to respond when the voltage was applied to the first sub-electrode. The oil ruptured in the area of sub-electrode 1 and moved toward the pixel corner. The oil rupture time was 0.7 ms. The aperture ratio of the pixel was 16.6% at 2 ms. Then, while keeping the first sub-electrode with continued voltage application, the same voltage was applied to two adjacent sub-electrodes to accelerate oil motion and promote oil to move rapidly in a specified direction. Aperture ratios of the pixel at 3 ms, 4 ms, and 5 ms were 38.9%, 47.5%, and 50.9%, respectively. The simulation results showed that it took only 4.7 ms for the proposed multi-electrode pixel structure to reach an aperture ratio of 50%, and the maximum aperture ratio was 57.9% at 9.5 ms.

**Figure 7:**
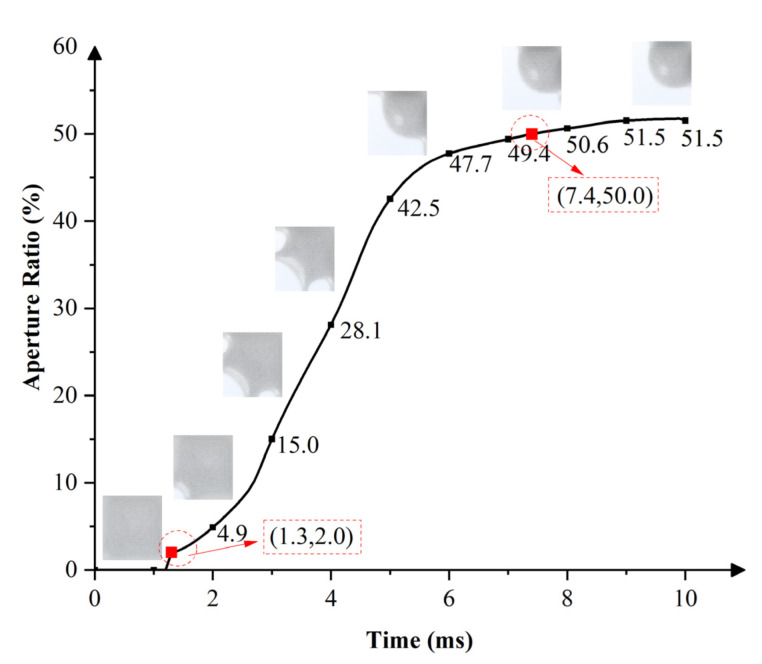
The switch-on response curve of an EWD pixel with a single-electrode pixel structure. The oil ruptured at 1.3 ms and the pixel reached a maximum aperture ratio of 51.7% at 9.5 ms, while 7.4 ms was required to reach an aperture ratio of 50%.

**Figure 8:**
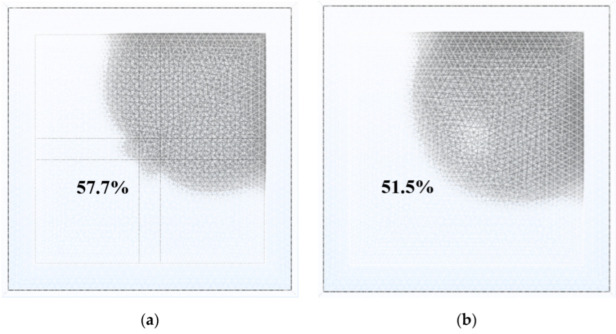
Maximum aperture of the multi-electrode pixel and the single-electrode pixel. (**a**) Top view of oil state when the multi-electrode pixel was fully opened. (**b**) Top view of oil state when the single-electrode pixel was fully opened.

Similarly, modeling and simulation were used to study the dynamic response of the EWD with a single-electrode pixel structure. Under the same boundary conditions, oil began to shrink and completely opened when the same voltage of 18 V was applied. The response curve of the single-electrode pixel opening process is shown in Figure 7. Oil began to rupture at 1.3 ms. The aperture ratio was 4.9% at 2 ms and 15% at 3 ms. Compared with the multi-electrode pixel, the aperture ratio reached 38.9% at 3 ms. In addition, at 9.5 ms, the single-electrode pixel achieved a maximum aperture ratio of 51.7%, but it was 6.2% lower than that of the multi-electrode pixel. Figure 8 shows the maximum aperture of two pixels. Oil rupture time was 0.6 ms slower than that of the multi-electrode pixel. Moreover, 7.4 ms was required for the single-electrode pixel to reach an aperture ratio of 50%, which was 2.7 ms slower than that of the multi-electrode pixel.

### 4.3. Effects of Pixel Electrode Structure on Oil Acceleration Capacity

**Figure 9:**
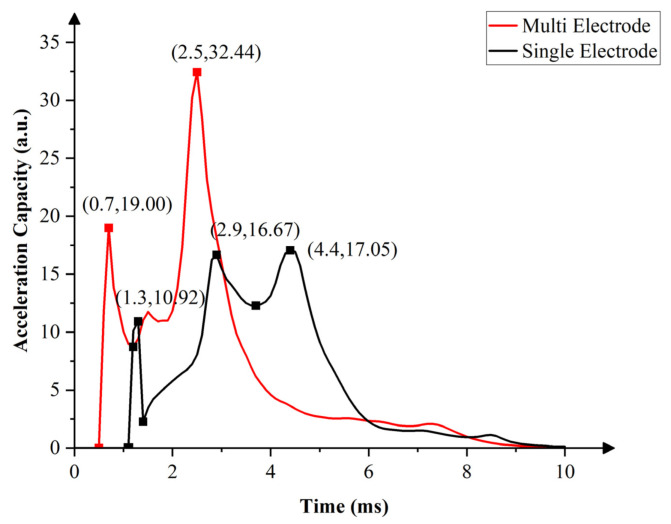
Comparison of oil-acceleration capability between the multi-electrode pixel and the single-electrode pixel. Three accelerations happened to achieve one switching response in the single-electrode pixel, whereas the multi-electrode pixel had two accelerations. For the multi-electrode pixel, peak values at its two accelerations were higher than the single-electrode pixel.

By deriving the function after fitting the response data in Figure 6; Figure 7, acceleration-capacity curves of oil in two different pixels were obtained, as shown in Figure 9. In the pixel opening process, oil in the multi-electrode pixel was accelerated twice, while oil in the single-electrode pixel was accelerated three times. This was because the multi-electrode could dynamically select the driving electrode according to the expected effect of oil motion. At 0.7 ms, oil of the multi-electrode pixels had achieved the first acceleration, but for oil in the single-electrode pixel, the first acceleration was achieved at 1.3 ms. Compared with the single-electrode pixel, oil in the multi-electrode pixel had a stronger response. In addition, the second acceleration occurred at 2.5 ms in the multi-electrode pixel structure, while oil in the single-electrode pixel structure was accelerated for the second and third times at 2.9 ms and 4.4 ms. In the process of multi-electrode pixel oil movement, peak values at its two accelerations were higher than that of the single-electrode. Oil in the multi-electrode pixel had higher acceleration capacity than the single-electrode pixel.

## 5. Conclusions

In this paper, a new pixel circuit structure with four sub-electrodes is proposed, and a three-dimensional dynamic simulation model of EWDs was established by COMSOL Multiphysics. The switching processes in the single-electrode and multi-electrode pixels were simulated. It was verified that the proposed multi-electrode pixel structure can effectively shorten the response time in the opening stage from theoretical and experimental perspectives. Hence, the multi-electrode pixel structure provides a novel method for improving the display effect of EWDs. This has significance for research and development of electrowetting devices.

## Figures and Tables

**Table 1:** Material, structure, and interface parameters used in the simulation.

Parameters	Quantity	Symbol	Value	Unit
Material	Density of oil	$\rho_{oil}$	900	kg/m^3^
	Density of water	$\rho_{water}$	1000	kg/m^3^
	Viscosity of oil	$\mu_{oil}$	0.004	Pa·s
	Viscosity of water	$\mu_{water}$	0.001	Pa·s
	Dielectric constant of oil	$\varepsilon_{oil}$	4	1
	Dielectric constant of water	$\varepsilon_{water}$	80	1
	Dielectric constant of hydrophobic dielectric layer	$\varepsilon_{hyd}$	1.287	1
	Dielectric constant of grid	$\varepsilon_{grid}$	3.28	1
Structure	Thickness of pixel	$w_{pixel}$	50	μm
	Width of pixel	$w_{pixel}$	160	μm
	Thickness of grid	$d_{grid}$	8	μm
	Width of grid	$w_{grid}$	15	μm
	Thickness of hydrophobic dielectric layer	$d_{hyd}$	1	μm
	Thickness of oil	$d_{oil}$	7	μm
Interfacial	Surface tension coefficient of oil and water	$\gamma_{ow}$	0.02	N/m
	Contact angle of grid	$\theta_{grid}$	140	deg
	Contact angle of hydrophobic surface	$\theta_{hyd}$	160	deg
	Contact angle of top substrate	$\theta_{top}$	30	deg
